# Dielectric Characterization of Breast Biopsied Tissues as Pre-Pathological Aid in Early Cancer Detection: A Blinded Feasibility Study

**DOI:** 10.3390/diagnostics13183015

**Published:** 2023-09-21

**Authors:** Eliana Canicattì, Daniel Álvarez Sánchez-Bayuela, Cristina Romero Castellano, Paul Martín Aguilar Angulo, Rubén Giovanetti González, Lina Marcela Cruz Hernández, Juan Ruiz Martín, Gianluigi Tiberi, Agostino Monorchio

**Affiliations:** 1 Department of Information Engineering, University of Pisa, 56126 Pisa, Italy; 2 Consorzio Nazionale Interuniversitario per le Telecomunicazioni (CNIT), 43124 Parma, Italy; 3 Free Space Srl, 56122 Pisa, Italy; 4 Breast Imaging Department, Radiology Service, Complejo Hospitalario Universitario de Toledo, 45007 Toledo, Spain; 5 Faculty of Chemical Science and Technology, Instituto Regional de Investigación Científica Aplicada, University of Castilla—La Mancha, 13001 Ciudad Real, Spain; 6 Anatomic Pathology Service, Complejo Hospitalario Universitario de Toledo, 45007 Toledo, Spain; 7 UBT—Umbria Bioengineering Technologies, 06081 Perugia, Italy; 8 School of Engineering, London South Bank University, London SE1 0AA, UK

**Keywords:** dielectric characterization, open-ended coaxial probe, breast biopsy, blinded pilot study, virtual transmission line model (VTLM)

## Abstract

Dielectric characterization has significant potential in several medical applications, providing valuable insights into the electromagnetic properties of biological tissues for disease diagnosis, treatment planning, and monitoring of therapeutic interventions. This work presents the use of a custom-designed electromagnetic characterization system, based on an open-ended coaxial probe, for discriminating between benign and malignant breast tissues in a clinical setting. The probe’s development involved a well-balanced compromise between physical feasibility and its combined use with a reconstruction algorithm known as the virtual transmission line model (VTLM). Immediately following the biopsy procedure, the dielectric properties of the breast tissues were reconstructed, enabling tissue discrimination based on a rule-of-thumb using the obtained dielectric parameters. A comparative analysis was then performed by analyzing the outcomes of the dielectric investigation with respect to conventional histological results. The experimental procedure took place at Complejo Hospitalario Universitario de Toledo—Hospital Virgen de la Salud, Spain, where excised breast tissues were collected and subsequently analyzed using the dielectric characterization system. A comprehensive statistical evaluation of the probe’s performance was carried out, obtaining a sensitivity, specificity, and accuracy of 81.6%, 61.5%, and 73.4%, respectively, compared to conventional histological assessment, considered as the gold standard in this investigation.

## 1. Introduction

Diagnosed in 2.3 million women and accounting for 685,000 deaths worldwide in 2020, breast cancer is the most common cancer in women worldwide and represents the leading cause of death [1]. Breast cancer management involves a comprehensive approach to diagnosing and treating this prevalent disease, in which early detection is crucial to reduce mortality and typically achieved through regular screening [2,3,4,5].

In the clinical management of breast cancer, a diagnostic approach is employed following tumor detection to confirm the presence of the disease. The gold standard for confirming the disease involves histological assessment, augmented by immunohistochemistry analysis, which provides insights into the molecular type of cancer and assists in guiding the therapeutic process [6,7]. Various biopsy sampling techniques are currently utilized in hospitals and clinics to extract samples of the suspicious findings identified through any imaging technique, including fine needle aspiration (FNA) biopsy, vacuum-assisted biopsy (VAB), and percutaneous excisional biopsy [8]. These excisional tissue sampling techniques are imaging-guided through ultrasound, mammography (stereotactic guidance), or MRI [9]. Nevertheless, several limitations regarding these techniques have been identified and discussed [9,10,11,12]. The literature highlights that the acquisition of biopsied tissues may be prone to errors or inaccuracies, associated to false negatives, due to the inherent challenges associated with the biopsy technique. Moreover, the waiting times for pathology results tend to be prolonged, particularly in hospital or diagnostic departments that experience a heavy workload. In this regard, technologies utilizing tissue dielectric behavior are being actively investigated for broader applications in breast cancer, including real-time tissue classification and microwave imaging [13,14].

Several techniques for measuring dielectric properties have been reported in the literature [15,16]. Among them, the open-ended coaxial probe stands out for its simplicity, non-destructive nature, and suitability for both ex vivo and in vivo measurements [17]. However, existing commercial probes have several limitations [18], such as the need for multiple probes to achieve broadband characterization, leading to high costs, and the complexity of calibration phases with open/short/load systems. To address these limitations, we previously designed and developed an optimized open-ended coaxial probe that represents a successful compromise between mechanical feasibility and the utilization of the virtual transmission line model (VTLM) reconstruction algorithm [19]. For instance, the utilization of an open-ended coaxial probe for the classification of normal and malignant tissues may provide valuable decision support in addition to traditional biopsy methods, as it enables real-time tissue characterization. By measuring the frequency-dispersive dielectric properties, it becomes feasible to delineate and characterize biological tissues, allowing for the differentiation between healthy and cancerous tissue in a succinct manner.

Extensive literature supports the distinction between healthy and cancerous tissues based on their dielectric properties, wherein cancerous tissues exhibit higher dielectric properties primarily attributed to increased water content [20,21,22,23]. Additionally, some research focused on the application of the open-ended coaxial probe for the dielectric characterization of healthy and malignant breast tissues, including also in vivo measurements in animal models [18,24,25]. Lazebnik et al. conducted a large-scale study of the dielectric properties of the breast tissues obtained both from cancer surgeries and breast reduction surgeries, utilizing a 0.5–20 GHz open-ended coaxial probe technique. They reported notable disparities in the relative permittivity and conductivity between cancerous and metastatic breast tissues compared to normal and healthy tissues [26,27,28]. Martellosio et al. presented a comprehensive analysis based on experimental measurements of over 220 tissue samples obtained during surgery (ex vivo), with measurements conducted within 3.5 h of tissue excision, to elucidate the differences between normal and tumorous breast tissues [29]. Moreover, significant dielectric contrast between normal and tumor breast tissue in vitro cell lines was observed in several studies [30,31,32]. Notably, all of these investigations employed surgically excised breast tissue samples previously diagnosed as malignant. Finally, the microwave characterization method using open-ended coaxial probes has been employed for other applications such as hepatic malignancies identification and skin lesions analysis, where machine learning has also been implemented [33,34,35].

In this work, a blinded pilot feasibility study is proposed for the clinical assessment of our custom-designed open-ended coaxial probe in the clinical setting of a multidisciplinary breast unit of a hospital. The primary objective is to investigate the potential clinical application of our probe as a supportive tool for pre-classifying biopsy samples based on their dielectric properties. This approach aims to provide valuable information to pathologists, enabling them to prioritize samples for further analysis through standard histological assessment. The study involves the dielectric characterization of 64 biopsy samples obtained from 15 patients at Complejo Hospitalario Universitario de Toledo, Spain, in the framework of a multicentric study to evaluate a new microwave-based imaging device, MammoWave (UBT Srl, Rivotorto, Perugia, Italy) [36,37]. By employing a blind experimental methodology, where both pathologists and radiologists are unaware of the dielectric characterization results, we can accurately assess the effectiveness of our classification method in facilitating early breast cancer detection.

The remainder of this paper is organized as follows: Section 2 provides details on the clinical investigation, the electromagnetic characterization set-up used for obtaining the dielectric parameters, and the experimental procedures conducted in the pilot study. Then, a section including all obtained results presents the findings of conventional histological tissues’ assessment and their corresponding electromagnetic characterization, including a comparison with existing literature data, and the outcomes of the presented rule-of-thumb for classifying the same tissues based on their dielectric parameters. Additionally, the statistical analysis, encompassing sensitivity, accuracy, and specificity, is provided to evaluate the applicability of our method in comparison to histopathology outcomes. Finally, a discussion and conclusions are presented.

### Problem Statement

The primary objective of the study was to explore the potential clinical application of our custom-designed open-ended coaxial probe as a supportive tool for pre-classifying biopsy samples based on their dielectric properties. Histological assessment of biopsied specimens is currently the standard procedure for diagnosing and classifying breast tissue abnormalities. Nonetheless, this procedure may be time-consuming and subject to interobserver variation, resulting in potential delays in treatment decisions due to pathology turnaround times and, sometimes, non-conclusive results.

**Figure 1:**
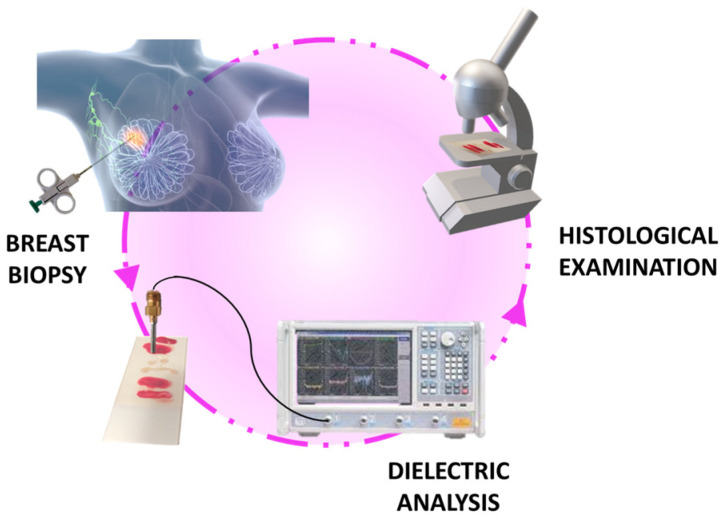
Research workflow, encompassing the biopsy procedure performed by radiologists, the analysis of samples using the open-ended coaxial probe, and the subsequent histological examination conducted by expert pathologists.

To address these issues, we investigated the feasibility of using dielectric characterization as an additional technique in supporting pathologists. Utilizing the different electromagnetic properties of biological tissues, dielectric characterization has the potential to provide valuable insights into tissue composition and distinguish benign from malignant samples. The research process followed a blinded methodology, ensuring objective and unbiased assessment. As depicted in Figure 1, biopsy samples were obtained from a cohort of patients and subsequently characterized using the open-ended coaxial probe. Measurements of the dielectric properties of breast tissues allowed for the reconstruction of their electromagnetic characteristics. The results of conventional histological examination, the gold standard for diagnosis, were then compared to these findings.

Using this methodology, the study intended to demonstrate the probe’s potential as a valuable instrument for real-time tissue classification. The ultimate goal is to support traditional biopsy methods by offering an objective and quantitative assessment of tissue properties, aiding pathologists in making informed decisions and prioritizing samples for further analysis. Properly using such a tool could improve the accuracy and efficiency of biopsy evaluations, resulting in more timely and accurate diagnoses and better patient outcomes.

## 2. Materials and Methods

### 2.1. The Clinical Investigation

The study was carried out in accordance with the principles enunciated in the current version of the Declaration of Helsinki, the guidelines of Good Clinical Practice (GCP) issued by ICH, the European Directive on medical devices 93/42/EEC, the corresponding ISO norms regarding clinical investigations of medical devices for human subjects and the application of risk management to medical devices [38,39], the European Law, and regulatory authorities’ requirements.

Clinical data used in this investigation were collected in the context of MammoWave clinical trials (Clinical Investigation to Evaluate the Ability of MammoWave in Breast Lesions Detection, ClinicalTrials.gov Identifier: NCT04253366), executed in Hospital Virgen de la Salud (Complejo Hospitalario Universitario de Toledo), Toledo, Spain, and approved by the corresponding Ethics Committee (id: 440); MammoWave informed consent was obtained from all subjects and/or their legal guardian(s).

### 2.2. Dielectric Properties Acquisition Set-Up

**Figure 2:**
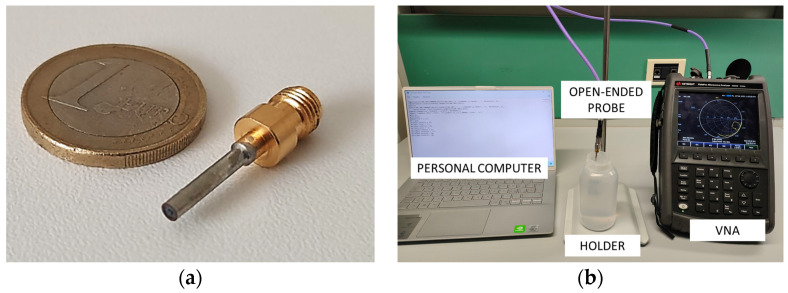
Open-ended coaxial probe prototype (**a**) and set-up consisting of an open-ended coaxial probe, a vector network analyzer (VNA), and an external computer (**b**).

The dielectric properties of the biopsy samples were measured using our custom-designed open-ended coaxial probe (Figure 2a). This probe consists of a section of a coaxial cable which is connected to a vector network analyzer (VNA) for the acquisition of the reflection coefficients (S11). Through appropriate inversion algorithms based on an equivalent analytical probe model, the tissues’ dielectric properties were reconstructed in terms of complex permittivity. Our probe was meticulously designed using an optimization procedure to minimize radiation losses and achieve a good compromise between physical feasibility and compatibility with the employed virtual transmission line model (VTLM) reconstruction algorithm [19]. This system allows for the accurate estimation of permittivity of materials having dispersive characteristics over a wide frequency range and is characterized by its speed and efficiency, utilizing only air and deionized water as calibration media. Unlike current commercial probes, our custom-designed probe is specifically tailored for broadband electromagnetic characterization (10 MHz–10 GHz), eliminating the need for expensive probe kits and cumbersome calibration processes with open/short/load-type systems. This makes our device highly feasible for clinical use and accessible for healthcare professionals.

The open-ended coaxial probe is composed of an inner dielectric, i.e., Teflon (PTFE), with a relative permittivity (ε′) of 2.1. The external diameter of the probe has been determined using the empirical criterion presented in [19] for selecting the radial aperture, resulting in a diameter of 1.52 mm. The small size of the probe ensures high accuracy in characterizing biological samples, making it suitable for detecting both healthy and malignant tissues.

In this experimental investigation, the reflection coefficients were obtained using a calibrated VNA (N9918A FieldFox, Keysight, Colorado Springs, CO, USA) within the frequency range 0.5–9 GHz (Figure 2b). To ensure accurate measurements, the coaxial probe was slightly pressed against the tissue samples, applying a controlled pressure to eliminate any air gap between the probe tip and the tissue, since it may adversely affect the measurements [18]. It has been observed in previous studies that the curvature radius affects the coupling between the probe and the tip, leading to inaccuracies in the reconstruction of the dielectric parameters [40,41,42]. However, by maintaining a minimal insertion depth of 0.3 mm, it is possible to achieve high precision and accuracy in the dielectric characterization of both healthy and malignant breast tissue using the presented probe. Consequently, the characterization data obtained from a 4 mm diameter excised tissue sample can be considered relevant and reliable for our study.

### 2.3. Experimental Procedure

**Figure 3:**
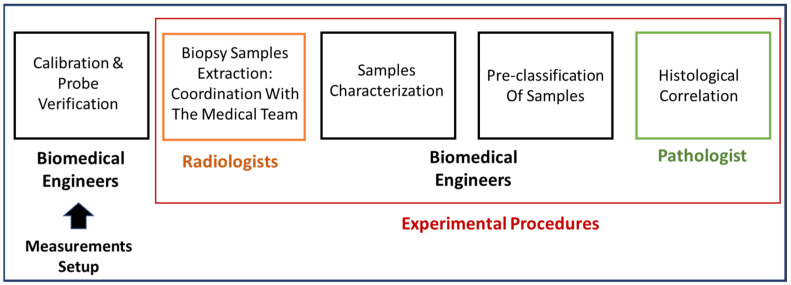
Sketch of experimental methodology followed during this investigation.

The experimental procedure followed the illustrated steps in Figure 3. The first step involved calibrating the coaxial probe using air and deionized water [19]. This calibration was fundamental to ensure accurate measurements of the reflection coefficient. By calibrating the probe in relation to the aperture plane, phase variation associated with its length was taken into account, resulting in a correction (de-embedding) process [43].

Once the acquisition set-up was prepared and the probe functionality was confirmed, the subsequent steps of the experiment were carried out in collaboration with the medical staff. The aim was to optimize the integration of electromagnetic measurements into routine clinical practice and minimize the time between tissue excision, dielectric measurement, and final handling of the samples for pathology analysis.

**Figure 4:**
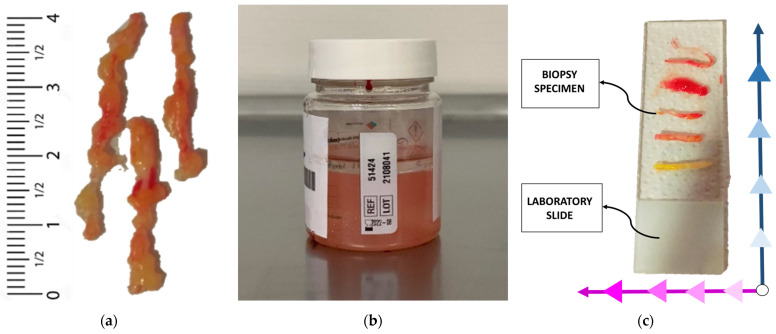
Examples of excised tissues’ size (**a**), excised tissue samples stored in formalin-filled container (**b**) and systematic acquisition approach (**c**).

In the presented pilot investigation, mammographic exams using Digital Breast Tomosynthesis (Selenia Dimensions, Hologic, Marlborough, MA, USA) were carried out on study volunteers; subsequently, the corresponding radiologist read the images and assigned a BI-RADS score to each suspicious lesion using the Breast Imaging Reporting and Data System (BI-RADS) guidelines [44]. If deemed appropriate by the clinical pathologist responsible, further tests, including other imaging modalities and biopsy procedures, were performed for pertinent diagnosis. Then, according to clinical guidelines, tissue samples were obtained by a radiologist using US-guided core needle biopsy or stereotactic (mammography-guided) vacuum-assisted biopsy and placed on laboratory slides. Typically, 5–6 tissue samples are obtained through core needle biopsy, which involves the excision of small cylindrical, individual samples. On the other hand, vacuum-assisted biopsy is used to extract a cluster of various samples that encompass the entire breast lesion. To prevent dehydration and tissue degradation, the time between sample excision and dielectric analysis was kept around 2–3 min. Initially, serum was used for tissue cleaning following standard practice. However, this practice was deemed invalid, as the serum on the tissue’s surface altered the reconstructed dielectric properties. Instead of measuring the tissue’s dielectric parameters, those of the serum were unintentionally captured. The size of the sample varied, as shown in Figure 4a. Nonetheless, their width and thickness were at least 4 and 20 mm, respectively, to ensure optimal coupling between the probe tip and the tissue under test.

For instance, immediately after tissue excision, electromagnetic characterization of the fresh samples was performed. Each sample was subjected to one or more dielectric measurements. If the sample size was small (below 5 mm), only one measurement was performed. However, if the sample was large enough, 2 or 3 measurements were obtained from different points. To maintain a systematic approach, the specimens were evaluated in a sequential order from the bottom-up of the laboratory slide and from right to left, as depicted in Figure 4c. After handling each tissue sample, the probe tip was carefully cleaned to prevent contamination.

Once the acquisitions were completed, the dielectric parameters (permittivity and conductivity) were reconstructed in real-time with our inversion algorithm. The obtained dielectric characterization results were then compared to existing dispersive models found in the literature [45,46]. To differentiate between healthy and tumor tissues, a percentage error formula was implemented as follows:

(1) $$\%\;Error=\left|\frac{V_{Calculated}-V_{Theoretical}}{V_{Theoretical}}\right|\times 100$$

where $V_{Theoretical}$ represents a quantity that has been proven or is widely accepted as true; specifically in our case, it refers to the theoretical literature dispersive dielectric properties of breast tissues. On the other hand, $V_{Calculated}$ represents a quantity derived numerically or experimentally, which in our work represents the dielectric properties of the breast tissues extracted using our dielectric characterization method. The percentage error measures the difference between the estimated value and the actual value, expressed as a percentage. This approach allows us to assess the magnitude of the error relative to the true value. Thus, in this study, we compare the dielectric parameters obtained from our open-ended coaxial probe with the theoretical literature values for the dielectric parameters of the breast tissues across the entire frequency range of interest.

To achieve optimal classification, a threshold of less than 20% (both for permittivity and conductivity) for the percentage error was set. This threshold was used to determine whether the tissue sample was healthy or malignant. The percentage error was calculated across the entire frequency range (0.5–9 GHz) between the dielectric properties extracted by our characterization method and literature data of healthy (adipose and fibroglandular) or tumor tissues. If the error value for either permittivity or conductivity with respect to the used dispersive models was below the 20% threshold, the tissue sample was classified as healthy or tumor accordingly. Specifically, based on the following criteria, a positive (P) or negative (N) score was assigned: If the presence of cancer for each sample is equal or greater than 50%, considering all analyzed points, the sample was classified as malignant or positive (P) and stored in container A. Otherwise, samples were stored in container B as benign tissues. If consistent results were obtained for multiple samples taken from the same patient (indicating either healthy or tumor tissue), they were placed in a single container (A or B). Tissue samples were immersed in formalin for preservation before pathology analysis (Figure 4b).

The choice of a 20% threshold was based on several considerations. Firstly, the threshold was set conservatively to ensure high precision and reliability in the classification. By allowing a significant margin of error, consistent and robust results were required to exceed this threshold. This approach minimizes the risk of misclassification of tissues and reduces the likelihood of diagnostic errors. Secondly, the threshold accounts for the inherent variability in the dielectric properties of tissues. Even within the same category (healthy or malignant), the dielectric parameters can vary due to individual patient differences, intra-tumoral heterogeneity, or limitations of the measurement techniques [47]. By allowing a wider threshold, the classification process accommodates this variability and ensures a more reliable and robust classification.

Once the electromagnetic characterization was completed and samples were labelled in the specific containers, they were sent to the pathology anatomy laboratory for conventional histological assessment. It is important to note that this study was conducted blindly, meaning the pathologist was unaware of the dielectric properties’ characterization results beforehand; the containers’ labelling (A or B) was kept unknown to the receiving pathologist responsible for the conventional histological assessment. The pathologist examined the contents of the labeled containers and produced a report in which his conventional histological outcomes were shown for each container. Finally, dielectric, and histopathological assessments were compared. Each step of the experimental procedures was carried out within the regular medical routine, and no additional biopsy procedures were conducted due to this experimentation.

## 3. Results and Discussion

### 3.1. Experimental Results

**Figure 5:**
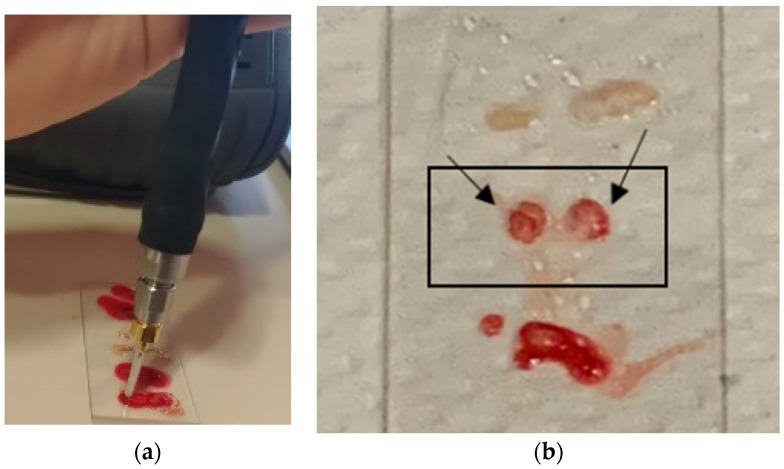
Example of experimental dielectric characterization: open-ended coaxial probe in contact with a tissue sample (**a**) and several excised tissue samples on laboratory slide (**b**).

All dielectric measurements were conducted at the Breast Imaging Department of the University Hospital of Toledo (formerly named Hospital Virgen De La Salud)—Complejo Hospitalario Universitario de Toledo, Spain. More precisely, biopsy specimens were measured immediately after excision, assuming their temperature was close to body temperature, while the measurement equipment (VNA and probe for open coaxial cable) had been set up in a temperature-controlled room (22 °C) to ensure the stability of the system heating and high measurement accuracy. Specifically, the dielectric parameters of excised breast tissues were derived by using a custom-designed open-ended coaxial probe connected to a VNA. The reflection coefficients were recorded with 1001 frequency points on a linear scale across the microwave frequency range of 0.5–9 GHz. Prior to conducting the electromagnetic characterization and analyzing the dielectric parameters, the probe system underwent calibration, as described earlier. Then, samples were dielectrically characterized via the open-ended coaxial probe, as depicted in Figure 5a. The collected data were then processed using our dedicated in-house algorithm and compared to the dispersive literature models. In total, 162 points from 64 excised breast tissue samples collected from 15 different volunteers were dielectrically characterized and included in the study. Tissues representing different breast lesions and radiological conditions, including both normal, healthy, and cancerous breast tissues, were gathered and analyzed.

Table 1 provides a summary of collected data and results of the study, including the following information for each subject: study identifier, the number of excised samples collected, the number of points dielectrically characterized per sample, the output from mammography radiological assessment, the output based on our proposed rule-of-thumb using dielectric characterization, and the gold standard output representing the histological outcome.

**Figure 6:**
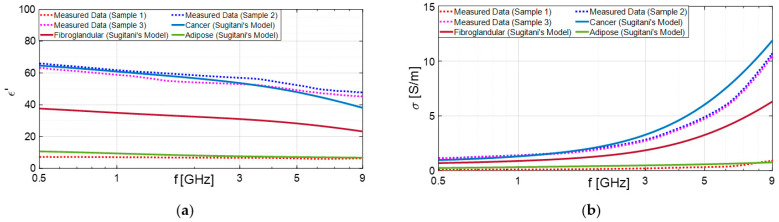
Dielectric properties: dielectric constant (**a**) and conductivity (**b**) extracted from excised tissues’ electromagnetic characterization of subject 10 and compared with Sugitani’s dispersive model [45].

As shown in Table 1, in most cases, the results of our classification obtained through open-ended coaxial probe are in good agreement with the corresponding pathology analysis. A notable example is the analysis of samples from subject 10, which demonstrates the successful functionality of the device (Figure 6). The dielectric characterization enables the differentiation between malignant and benign lesions, and the results are consistent with the literature data for the respective malignant lesion and adipose tissue.

However, there were instances where false positives occurred, meaning samples were classified as malignant lesions but were ultimately determined to be benign. Some authors have highlighted the substantial variation in the frequency response of cancerous tissue samples due to the non-homogeneity of breast tissue [27,48]. Different pieces of tissue may contain varying proportions of adipose, fibroconnective, and glandular tissues. The complex permittivity has an average variation between high water content healthy and cancerous tissue of only about 10% [27,48]. Consequently, distinguishing between them becomes challenging.

According to Sugitani et al., adipose tissues have a dielectric constant approximately four times lower than that of cancerous tissues [45]. As a result, they can be effectively distinguished, as demonstrated in the previous case shown in Figure 6. On the other hand, the differences between cancer and fibroglandular tissues are not significant. In fact, the dielectric constant of cancerous tissue (ranging from approximately 35 to 65) are very similar to those of fibroglandular tissues (ranging from 15 to 50). This similarity may elucidate why fibrosis or fibroadenoma was misidentified as carcinoma through dielectric characterization, as observed in subjects 3 and 16, for example.

**Figure 7:**
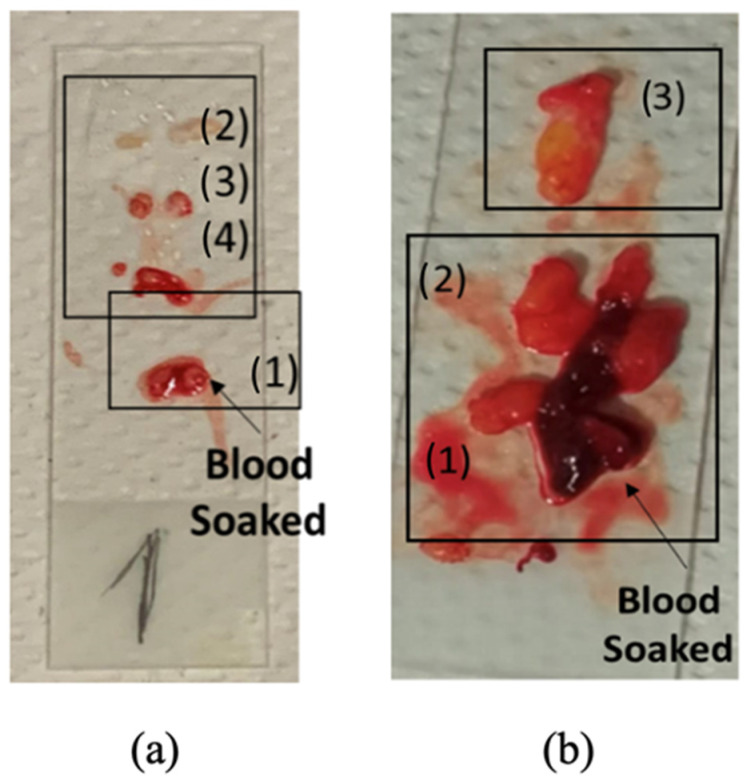
Excised breast tissue samples extracted using VAB placed on laboratory slides: subject 3b (**a**) and subject 15 (**b**).

**Figure 8:**
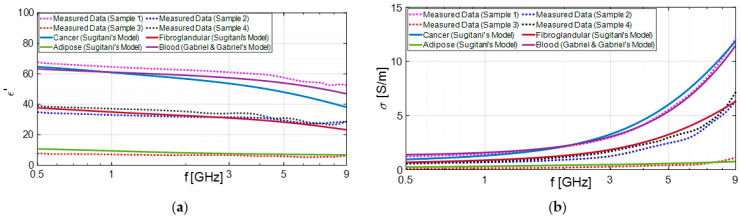
Dielectric properties: dielectric constant (**a**) and conductivity (**b**) extracted from excised tissues’ electromagnetic characterization of subject 3b and compared with Sugitani’s and Gabriel’s dispersive models [45,46].

**Figure 9:**
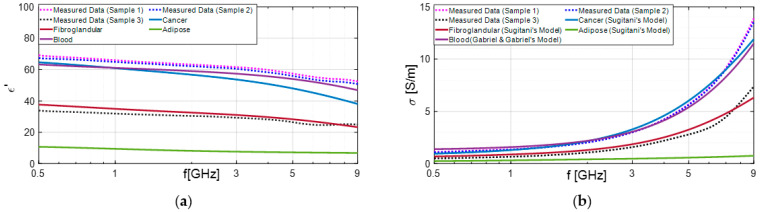
Dielectric properties: dielectric constant (**a**) and conductivity (**b**) extracted from excised tissues’ electromagnetic characterization of subject 15 and compared with Sugitani’s and Gabriel’s dispersive models [45,46].

Furthermore, we verified that most of the malignant samples that were ultimately identified as false positives were soaked in blood, as illustrated in Figure 7. Notably, the results obtained from subjects 3 and 15, depicted in Figure 8 and Figure 9, respectively, are noteworthy. We compared these findings with the dielectric properties of blood and found them to be quite similar to those of cancerous samples [46]. Consequently, distinguishing between the two becomes challenging.

### 3.2. Statistical Analysis

The sensitivity and specificity of a decision criterion can be used to describe its diagnostic performance. Sensitivity, also referred to as the true positive (TP) rate, indicates the percentage of breast cancer cases accurately diagnosed with the disease. A false negative (FN) occurs when a patient with a malignant lesion (in our case, breast cancer) receives a negative diagnosis. Specificity, also known as the true negative (TN) rate, represents the percentage of healthy cases correctly identified as not having breast cancer. Finally, a false positive (FP) is defined when the test indicates the presence of the disease, but the patient does not actually have it. For instance, sensitivity, specificity, and accuracy are related to the TP, FP, TN, and FN values [49,50]:

(2) $$Sensitivity=\frac{TP}{TP+FN}$$

(3) $$Specificity=\frac{TN}{TN+FP}$$

(4) $$Accuracy=\frac{TP+TN}{TP+TN+FP+FN}$$

This statistical analysis enabled us to evaluate the effectiveness of our proposed method in detecting the presence or absence of breast cancer in tissues samples. Specifically, we conducted a sensitivity and specificity analysis by comparing the outcomes of the presented rule-of-thumb with those of the pathologists, as histological assessment is considered the gold standard in diagnosing breast cancer. The collected data were processed using MatLab and Excel spreadsheets.

As depicted in Table 2, the confusion matrix was computed, which indicates the number of cases correctly or incorrectly classified. The calculated results for our characterization system compared to the gold standard are as follows: sensitivity of 81.6%, specificity of 61.5%, and accuracy of 73.4%. In a study conducted by Martellosio et al., the real part of the dielectric permittivity was utilized to discriminate between normal and tumor tissues by selecting specific cut-off values at two frequencies (1 and 50 GHz). At 1 GHz, the achieved sensitivity was 92.6%, and specificity was 72.7%. On the other hand, at 50 GHz, the obtained sensitivity and specificity were 71.4% and 66.8%, respectively [29].

As aforementioned, our gold standard was the conventional histological assessment. The performance of the presented open-ended coaxial probe system exhibits high sensitivity and moderate specificity. The high sensitivity can likely be attributed to two factors: the small diameter of the probe and its optimal design in terms of length. In fact, the probe was specifically designed to reduce radiation losses. As a result, the occurrence of the fringing field effect in air can be avoided, allowing for optimal electric field confinement [41]. Consequently, the measurement of the reflection coefficient becomes more accurate, leading to improved reconstruction of the dielectric properties. Instead, the moderate specificity could be probably related to the similarity between blood and cancer dielectric properties [27].

### 3.3. Limitations

This investigation has some limitations. Specifically, using a threshold (predetermined cut-off) in rule-of-thumb approaches for diagnosis may lead to the following: (1) a lack of individual variability, as individual subjects may have unique characteristics and variations in their tissues’ dielectric parameters, (2) overgeneralization as cancer types, age groups, or demographic factors can influence the optimal threshold for diagnosis, making it challenging to apply a single rule-of-thumb across diverse populations, (3) limited specificity to differentiate between similar conditions due to false positives, and, summarizing, a lack of personalized approach as rule-of-thumb thresholds are inherently standardized and may not consider individual patient characteristics, comorbidities, or personalized factors that influence disease diagnosis.

Future developments will be devoted to improving the performance of the system and developing strategies to approach personalized medicine, tailoring breast tissues’ classification to individual patients. As an example, IoT could enable us to gather real-time data from connected medical devices and the adaptation of diagnostic thresholds in a more personalized manner [51].

## 4. Conclusions

This feasibility study assessed the initial use of a functional open-ended coaxial probe to discriminate between healthy and malignant breast tissues. These methods may have a wide range of clinical applications in different fields and settings as they are simple to use, do not require dedicated calibrations or specific sample manipulation, and provide rapid and inexpensive tissue pre-classification when quick characterization results are required.

Achieving optimum accuracy in tissue classification may offer probe-based systems interesting applications in clinical settings that should be investigated in prospective, dedicated trials. Specifically, this kind of device may aid in prioritizing histological and immunohistochemical assessments of samples with high chances of being malignant according to their dielectric parameters. Furthermore, dielectric characterization may facilitate the evaluation of benign lesions, thereby reducing patients’ stress and unnecessary expenses associated with additional examinations. Lastly, real-time applications in tissue discrimination may be relevant in surgery, especially for determining optimum margins for tissue excision, allowing conservative surgeries and health tissue preservation.

## Figures and Tables

**Table 1:** Subjects’ details, dielectric characterization, and histopathology outcomes.

Subject ID	Number of Excised Tissue Samples	Number of Points Analyzed	Mammographic Lesion’s BI-RADS Score	Probe Rule-of-Thumb Output	Histological Outcomes
1	2	3	4A	Both samples positive (P)	No evidence of neoplasia (Benign)
2	6	11	4B	3 positive (P) and 3 negative (N) samples	Invasive ductal carcinoma, luminal (Malignant) in all analyzed samples
3a (Left breast, inner quadrant)	4	10	4B	1 positive (P) and 3 negative (N) samples	Fibrosis (Benign) in all analyzed samples
3b (Left breast, outer quadrant)	4	6	4B	2 positive (P) and 2 negative (N) samples	Fibrosis (Benign) in all analyzed samples
4	5	11	5	All samples positive (P)	Invasive ductal carcinoma (with in situ component) grade II, Her2-
5	3	7	5	All samples positive (P)	Invasive ductal carcinoma grade II, luminal
6	3	10	5	All samples positive (P)	Invasive ductal carcinoma (with in situ component) grade II, Her2+
7	2	16	3	All samples negative (N)	Stromal collagenization compatible with stromal hyperplasia
8	3	6	3	All samples negative (N)	Benign fibroepithelial lesion in all analyzed samples
9	5	13	5	All samples positive (P)	Invasive ductal carcinoma (with in situ component) grade II
10	3	8	5	2 positive (P) and 1 negative (N) samples	Invasive ductal carcinoma grade II in (A), adipose tissue in (B)
11	5	12	5	All samples positive (P)	Invasive ductal carcinoma grade II, intermediate luminal
12a (Right Breast)	3	8	5	1 positive (P) and 2 negative (N) samples	Microinvasive carcinoma grade I, luminal A
12b (Left Breast)	3	6	5	2 positive (P) and 1 negative (N) samples	Microinvasive carcinoma grade I, luminal A
13	7	20	4B	2 positive (P) and 5 negative (N) samples	Adenosis and mastopathy in all analyzed samples
14	3	7	4B	2 positive (P) and 1 negative (N) samples	Invasive ductal carcinoma grade II, luminal A in all analyzed samples
15	3	8	5	3 positive (P) samples	Fibroadenoma in all analyzed samples

**Table 2:** Confusion matrix used in our breast biopsy tissue classification method.

		Status of Patient According to “Gold Standard (Histological Assessment)”	
		Presence of Malignant Lesions	Absence of Malignant Lesions
Presented Rule-of-Thumb Output	Positive	TP 31	FN 7
	Negative	FP 10	TN 16

## Data Availability

There are no restrictions about data sharing. The minimal anonymized dataset necessary to replicate our study findings is openly available in the following stable, public repository: London South Bank University, UK.
